# Supplementary material for: Nuclear enlargement induced by overexpression of nuclear export signal is associated with abnormal nuclear division in Schizosaccharomyces pombe
Source: Biol Open. 2025 Dec 3;14(11):bio062331. doi: 10.1242/bio.062331 (PMC12714134; doi:10.1242/bio.062331)
Supplement: Supplementary information [file biolopen-14-062331-s1.pdf]

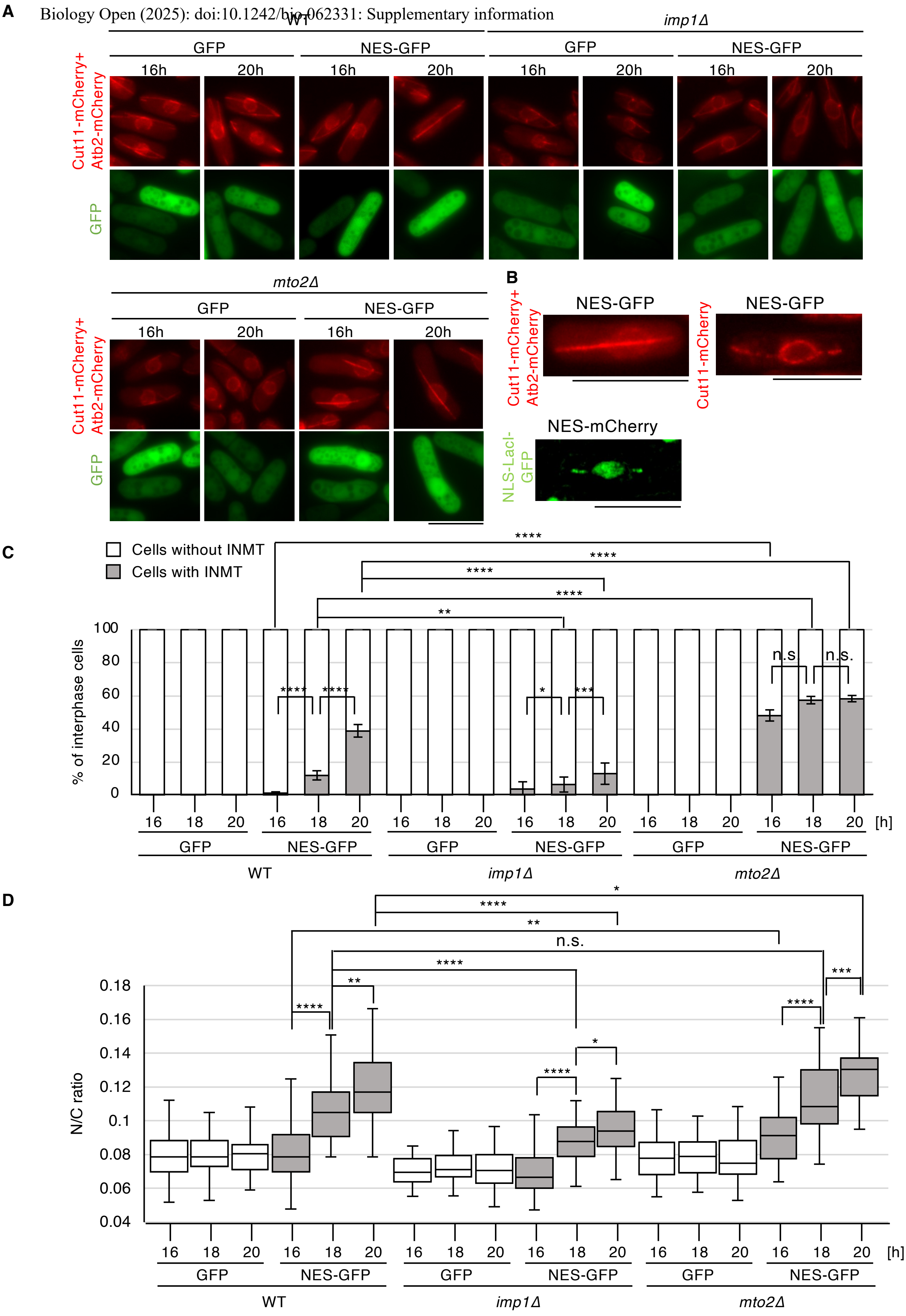

Fig. S1.
(A) Nuclear envelope (Cut11-mCherry) and GFP or NES-GFP of wild-type, *imp1Δ* and *mto2Δ* cells overexpressing GFP or NES-GFP. Cells were grown in EMM medium at 28°C. Scale bar: 5μm. (B) Both microtubule (Atb2-mCherry) and nuclear envelope (Cut11-mCherry) (Top, left), only nuclear envelope or Nucleoplasm (NLS-LacI/lactose repressor protein-GFP) of an interphase cell overexpressing NES-GFP or NES-mCherry. (C) The frequency of interphase cells with or without intranuclear microtubule bundles (INMT) (n = 50). Wild-type, *imp1Δ* and *mto2Δ* cells overexpressing GFP or NES-GFP were grown in EMM medium at 28°C. Statistical significance was assessed using Chi-square test. $**p<0.01$. $****p<0.0001$.(C) N/C ratio of wild-type, *imp1Δ* and *mto2Δ* cells overexpressing GFP or NES-GFP (n = 50). Cells were grown in EMM medium at 28°C. Statistical significance was assessed using Welch's t test. $*\ p<0.05$, $**\ p<0.01$, $***p<0.001$, $****p<0.0001$.

Biology Open • Supplementary information

Table S1. N/C ratio, nuclear volume and cell volume of WT, *imp1Δ* and *mto2Δ* cells overexpressing GFP or NES-GFP (Data of Fig. S1)

			N/C ratio		Nuclear volume (μm^3)		Cell volume (μm^3)	
WT	GFP	16h	0.079	± 0.013	10.0	± 1.8	128	± 24
		18h	0.081	± 0.012	9.7	± 1.6	121	± 18
		20h	0.080	± 0.012	8.8	± 1.7	110	± 16
	NES-GFP	16h	0.083	± 0.018	9.9	± 2.5	120	± 21
		18h	0.106	± 0.018	15.0	± 3.7	143	± 35
		20h	0.120	± 0.020	17.9	± 7.2	150	± 53

			N/C ratio		Nuclear volume (μm^3)		Cell volume (μm^3)	
imp1Δ	GFP	16h	0.070	± 0.008	7.8	± 1.3	112	± 18
		18h	0.072	± 0.009	7.9	± 1.3	110	± 17
		20h	0.074	± 0.016	7.5	± 1.5	104	± 22
	NES-GFP	16h	0.071	± 0.016	8.3	± 1.6	121	± 22
		18h	0.089	± 0.014	10.0	± 2.0	115	± 25
		20h	0.095	± 0.015	11.9	± 3.0	126	± 27

			N/C ratio		Nuclear volume (μm^3)		Cell volume (μm^3)	
mto2Δ	GFP	16h	0.079	± 0.013	11.9	± 2.3	154	± 37
		18h	0.079	± 0.012	10.6	± 1.8	136	± 23
		20h	0.078	± 0.014	10.4	± 1.7	135	± 23
	NES-GFP	16h	0.091	± 0.014	16.4	± 4.0	181	± 34
		18h	0.118	± 0.029	20.5	± 5.5	179	± 48
		20h	0.129	± 0.018	24.3	± 5.8	190	± 41

Table S2. N/C ratio, nuclear volume and cell volume of WT cells overexpressing GFP or NES-GFP (Data of Fig.3B)

			N/C ratio		Nuclear volume (μm^3)		Cell volume (μm^3)	
WT	GFP	Normal division	0.079	± 0.015	14.5	± 3.1	184	± 27
	NES-GFP	Normal division	0.096	± 0.016	17.7	± 7.9	179	± 52
		Fig. 2C	0.112	± 0.020	23.5	± 9.4	205	± 48
		Fig. 2D	0.112	± 0.004	19.9	± 2.8	178	± 19

Biology Open • Supplementary information

Table S3. Strain list

Strain name	Genotype
TF2769-4a	*h- cut11^{+}:mCherry:natr leu1 pREP1-GFP*
TF2769-3a	*h- cut11^{+}:mCherry:nat^{r} leu1 pREP1-NES-GFP*
TF487-4a	*h+ Z::P$_{adh15}$:mCherry:atb2:nat^{r} cut11^{+}:mCherry:nat^{r} leu1 ura4 pREP1-GFP*
TF487-3a	*h+ Z::P$_{adh15}$:mCherry:atb2^{+}:nat^{r} cut11^{+}:mcherry:nat^{r} leu1 ura4 pREP1-NES-GFP*
TF1034-4a	*h? imp1Δ::kanr Z::P$_{adh15}$:mCherry:atb2:nat^{r} cut11^{+}:mCherry:nat^{r} leu1 ura4 pREP1-GFP*
TF1034-3a	*h? imp1Δ::kanr Z::Padh15:mcherry:atb2:natr cut11^{+}:mCherry:nat^{r} leu1 ura4 pREP1-NES-GFP*
TF952-4a	*h? mto2Δ::kanr cut11^{+}:mCherry:nat^{r} Z:P$_{adh15}$:mCherry:atb2^{+}:nat^{r} leu1 pREP1-GFP*
TF952-3a	*h? mto2Δ::kanr cut11^{+}:mCherry:nat^{r} Z:P$_{adh15}$:mCherry:atb2^{+}:nat^{r} leu1 pREP1-NES-GFP*
TF2772-3a	*h^{-} NLS-LacI-GFP cut11^{+}:mCherry:nat^{r} leu1-32 pREP1-NES-mCherry*

Table S4. Oligo list

Plasmid (pREP1-NES-GFP) construction (forward)	AAGTCGACTATGGAATTAGCCTTGAAATTA
Plasmid (pREP1-NES-GFP) construction (reverse)	AAAGATCTTTATTTGTACAATTCATCCATA
Plasmid (pREP1-GFP) construction (forward)	AAGTCGACTATGTCTAAAGGTGAAGAATTA
Plasmid (pREP1-GFP) construction (reverse)	AAAGATCTTTATTTGTACAATTCATCCATA

Biology Open • Supplementary information
